# The Li_2_SO_4_–Na_2_SO_4_ System for Thermal Energy Storage

**DOI:** 10.3390/ma12223658

**Published:** 2019-11-07

**Authors:** Stefania Doppiu, Jean-Luc Dauvergne, Angel Serrano, Elena Palomo del Barrio

**Affiliations:** 1 CIC energiGUNE, 01510 Vitoria-Gasteiz, Spain; jldauvergne@cicenergigune.com (J.-L.D.); aserrano@cicenergigune.com (A.S.); epalomo@cicenergigune.com (E.P.d.B.); 2 Ikerbasque, Basque Foundation for Science, 48013 Bilbao, Spain

**Keywords:** solid state reactions, solid-state phase transitions, thermal energy storage, energy density, ball milling

## Abstract

In this paper, the system Li_2_SO_4_–Na_2_SO_4_ is proposed as a candidate material for thermal energy storage applications at high temperatures (450–550 °C). Depending on the composition, the thermal energy can be stored by using a eutectoid reaction and solid–solid phase transition. In these types of systems, all the components (reagent and products) are in the solid state. This work includes the theoretical analysis (based on the Calphad method) of the system selected obtaining all the theoretical parameters (for example, enthalpies of reaction, transition temperatures, volume expansion, and the heat capacities) necessary to determine the theoretical performance in terms of thermal energy storage. The theoretical analysis allowed to identify two compositions (Li_2_SO_4_/Na_2_SO_4_ 79/21 and 50/50) in the phase diagram with the most promising theoretical enthalpy of transformation (270 J/g and 318 J/g, respectively) corresponding to a eutectoid reaction and a solid–solid phase transition (stoichiometric compound LiNaSO_4_). The experimental analysis carried out allowed to confirm the great potential of this system for TES application even if some discrepancies with the theoretical calculation have been observed experimentally (energy densities lower than expected). For the two compositions studied, 79/21 and 50/50, the enthalpies of reaction are 185 J/g and 160 J/g, respectively. The reactivity of the system was tested under different experimental conditions preparing materials with a different degree of nanocrystallization to favor the diffusion in the solid state, testing the reactivity of the materials under controlled atmosphere and under air, and performing preliminary durability analysis (cycling behavior up to 20 cycles) to test the stability and reversibility.

## 1. Introduction

In a previous paper, we reported the great potential of using solid state reactions for the storage of thermal energy [1]. In the paper, we present a list of possible reactions (over a wide range of temperatures) with promising energy densities for the application in thermal energy storage (TES). The use of these typologies of reactions would allow to build “simple”, compact and low-cost thermal energy storage systems that could be used in different applications, such as solar, heat in industrial processes, power generation, waste heat recovery, etc. The need to store, among other types of energy, the thermal energy comes from the necessity to decarbonize our society and promote the development of renewable energy sources able to supply energy constantly, without interruption, and able to face the demand of energy in the peak periods [2,3]. Storing thermal energy will help to the penetration and dispatchability of renewable energies and will contribute to creating a low carbon society for environmental protection increasing energy efficiency and decreasing energy demand [4,5,6]. When comparing to other types of thermal storage (sensible, latent and thermochemical [7,8,9,10]), the use of solid state reactions (belonging to the thermochemical storage class) and solid–solid phase transitions (belonging to the latent heat storage class) presents many advantages, one above the others being the possibility to conceive thermal energy storage systems considerably simpler than the ones used in the case of gas–solid reactions or solid–liquid phase transition. Indeed, in the first case, two reactors are necessary to keep the reactants separated up to the moment when the discharging of the thermal energy is required, while, in the second case, most of the times, it is necessary to resort to encapsulation techniques to avoid leaks of the material and allow a direct contact with the heat transfer fluid [11].

Li_2_SO_4_–Na_2_SO_4_ is a promising system for solid state thermal energy storage. This system was already proposed by Chen et al. [12] as a possible material for TES application with the study (structural and thermal) of three main compositions in the phase diagram. Moreover, Li_2_SO_4_–Na_2_SO_4_ system has been extensively investigated due to its properties as an ionic conductor belonging to the solid electrolyte family. In particular, the composition corresponding to the stoichiometric compound LiNaSO_4_ (lithium-sodium sulfate) is a well-known superionic conductor, which has a quasi-liquid cationic sub-lattice above 515 °C [13]. This material has been extensively studied both structurally, with the determination of the structural transformations by XRD analysis and in situ-XRD during heating [14], thermally with the determination of the enthalpy, the entropy the specific heat of the transformation, the thermal conductivity [15], the thermal expansion [16] and spectroscopically with different techniques such as NMR [17,18], Raman [19,20,21] and IR [22]. Moreover, deep studies relevant to the kinetics of the phase transition have been carried out enlightening the fast kinetics of the transformation characterized by a low energetic barrier for the transformation α ↔ β. The results show that the “phase transition in LiNaSO_4_ is governed by the diffusion-controlled growth of germs, i.e., the limiting stage of the transition kinetics is related to the diffusion of cations.” [23].

In this study, two compositions in the system Li_2_SO_4_–Na_2_SO_4_ are considered as candidate materials for thermal energy storage at high temperatures (450–550 °C): 79/21 and 50/50 (molar ratio), corresponding to a eutectoid reaction and a solid–solid phase transition. Compared with the previous work of Chen et al. [12], this paper focusses on several key issues for the usability of a phase change material (PCM) in heat storage applications. A suitable PCM must not only have a high phase transition enthalpy, but must also be hysteresis-free, stable to cycling and insensitive to heating/cooling rates. Moreover, the compatibility of the storage material with air is an additional asset. On the one hand, this facilitates material handling and processing. On the other hand, in applications using air as heat transfer fluid, it can simplify the integration of the material into the storage system allowing direct contact with air, thus lowering cost. This paper deals with all above-mentioned aspects. Moreover, the possibility of increasing the reactivity of the studied materials is also investigated. In order to determine the relationship between structure and reactivity, different samples were prepared, subjecting the pure materials (Li_2_SO_4_ and Na_2_SO_4_) to different mechanical treatments (ball milling) with the attempt to obtain powders with different microstructures. The reactivity was then studied by differential scanning calorimetry analysis. The performance as TES materials was also tested, carrying out preliminary durability tests and evaluating the reactivity in air. As a result of this study, this system seems very promising for application in thermal energy storage due to high transition energies, good reversible behavior, the possibility to work in air, and the solid nature of the components.

## 2. Materials and Methods

### 2.1. Theoretical Evaluation and Materials Preparation

The theoretical phase diagram of the Li_2_SO_4_–Na_2_SO_4_ system was established by using the CALPHAD (CALculation of PHAse Diagram) method. Regular and sub-regular solution models were used to obtain the Gibbs energy functions of various solution phases. The excess Gibbs energy of each phase was represented by the Redlich–Kister formalism, with binary interaction parameters following the form of power series [24,25,26]. The parameters of these models were determined by using the optimization module of FactSage7.3 software (GTT-Technologies, Herzogenrath, Germany) [27] with the set of evaluated and optimized the thermodynamic database for inorganic anhydrous salts. All the key thermodynamic properties (e.g., enthalpy of reaction, specific heats, densities, volume change during the charge/discharge process) of identified eutectoid (Li_2_SO_4_–Na_2_SO_4_ 79/21) and solid-state phase transition (Li_2_SO_4_–Na_2_SO_4_ 50/50) were obtained assuming equilibrium conditions.

Li_2_SO_4_ and Na_2_SO_4_ anhydrous powder were supplied by Alfa Aesar with purities of 99.7% and 99%, respectively. The pure powders were placed in an Argon glove box (Brown) with levels of oxygen and humidity lower than 0.1 ppm. This was necessary in order to avoid the hydration of Li_2_SO_4_ achieving high precision when preparing the materials with a right stoichiometric ratio. The pure materials (Li_2_SO_4_ and Na_2_SO_4_) were subjected to mechanochemical treatments (Ball milling) in order to maximize the reactivity in the solid state to achieve powders with a controlled microstructure. The goal was to prepare highly reactive materials (high number of defects, high specific surface area, and high contact area) and activate the reaction subsequently by thermal treatment to determine the relationship between microstructure and reactivity.

For this purpose, a Spex mixer mill (875 RPM, Spexsampleprep, Metuchen, NJ, USA), using stainless steel vials and balls, was used. Different batches of pure salts were milled for 2, 4, and 8 h with a ball to powder mass ratio (BPR) of 1.6. The BPR was kept low in order to minimize the possible contamination coming from the milling media during the mechanical treatment. Subsequently, the two salts (milled for the same time) were mixed in the proper stoichiometric ratio (Li_2_SO_4_/Na_2_SO_4_ 79/21 and 50/50) and subjected to mechanical treatment under mild conditions (for 15 min using three balls of 3 g) to obtain a homogeneous mixture with a good intermixing degree.

### 2.2. Synthesis

The powder mixtures prepared by ball milling were pressed in the form of pellet subjected to a pressure of three tons for 5 min. A pellet die of 12 mm was used. The weight of each sample was around 0.3 g. The pellets were subjected to thermal treatment in an oven inside a specially designed stainless-steel reactor allowing to perform the thermal treatment under controlled atmosphere (argon). To test the reactivity in air similar experiments were carried out with the sample directly placed inside the oven (above an Al_2_O_3_ support) performing the heating treatment under air. All the samples were subjected to a heating treatment of up to 550 °C with a heating rate of 10 K/min and a free cooling rate (around 2 K/min as measured by a thermocouple placed inside the oven in the proximity of the sample).

### 2.3. Thermodynamic Characterization

The reactivity of the materials was tested by differential scanning calorimetry (DSC) technique using a Thermal Analysis Q2000 model (TAinstruments, New Castle, PA, USA). This technique allowed to determine the enthalpies of reaction/phase transition, the reaction temperatures, the heat capacities, and the behavior upon multiple cycles (up to 20) between 430 and 550 °C, including isothermal steps of 15 min between subsequent heating and cooling steps. To test the reactivity of the materials under different experimental conditions, different heating and cooling rates were applied (2, 5, 7.5, 10, 15, 20, 25, and 30 K/min). The heat capacities measurements were performed directly using the modulated heating ramp dynamic method. The selected continuous heating rate was 2 K/min. The instrument was previously calibrated using sapphire as standard material. The structural changes of the materials before and after DSC experiments were determined by XRD analysis.

### 2.4. Structural Analysis

The structural analysis of the materials (i) before milling, (ii) after milling, (iii) after synthesis in the oven, and (iv) after DSC was performed by X-ray diffraction analysis using a Bruker D8 Discover () equipped with a LYNXEYE XE detector with monochromatic Cu Kα1 radiation of λ = 1.54056 Å. Patterns were recorded in a 2θ angular range of 10–120° with a step size of 0.02° and a step time of 1.5 s. The measurements were performed at room temperature. A full profile fitting procedure of the diffraction patterns [28] based on the Rietveld method [29] was used to gain information about the phases formed and their relative percentages, the crystallite sizes, the microstrain level, and the structural parameters (cell parameters, atomic positions). The morphology of the material was studied by scanning electron microscopy (SEM,) using a Quanta 200 FEG (FEI Company, Hillsboro, OR, USA) scanning electron microscope operated in high vacuum mode at 30 kV and with a backscattered electron detector (BSED). In addition, energy-dispersive X-ray spectroscopy (EDX) analyses were carried out in order to obtain chemical composition maps.

## 3. Results and Discussion

### 3.1. Theoretical Analysis Results

**Figure 1:**
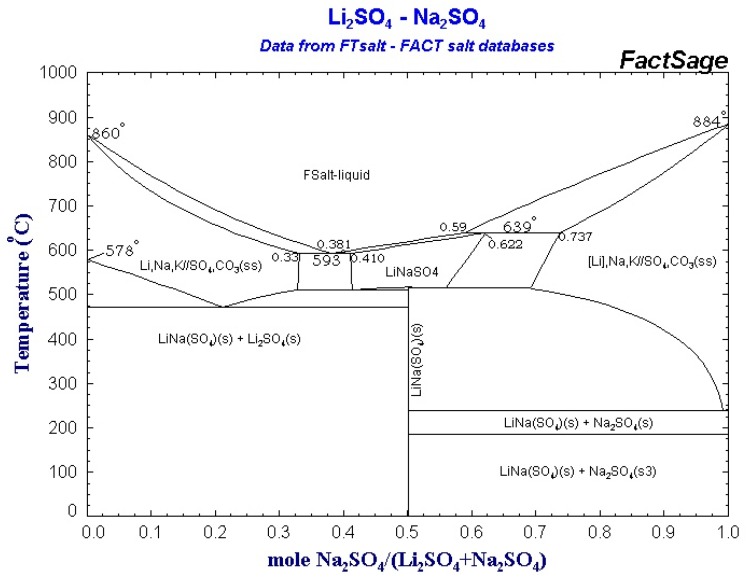
The theoretical phase diagram obtained using the FactSage 7.3 software.

In Figure 1 the theoretical phase diagram of the Li_2_SO_4_–Na_2_SO_4_ system obtained using the FactSage software is reported.

Analyzing the available experimental literature data, some discrepancies can be detected concerning the position of the eutectoid reaction (see Equation (1)).

The Calphad method (Figure 1) predicts the eutectoid reaction at the composition Li_2_SO_4_–Na_2_SO_4_ 79/21 This result is similar to the experimental one obtained by Schroeder et al. [30] but differs from the results of Mata et al. [21] and Nacken et al. [31] where the eutectoid reaction is placed at the composition Li_2_SO_4_/Na_2_SO_4_ 72.6./27.4. In this study, following the theoretical results obtained using the Calphad method, the compositions 79/21 and 50/50, with the highest theoretical energy densities were investigated experimentally.

The eutectoid reaction corresponding to the composition Li_2_SO_4_/Na_2_SO_4_ 79/21 is reported below:

β-Li_2_SO_4_ + β-LiNaSO_4_ ↔ α-Li_2_SO_4_ (ss) (1)

In this reaction, the β-Li_2_SO_4_ (monoclinic structure) and the stoichiometric compound β-LiNaSO_4_ (trigonal structure) react (at the proper temperature) forming the solid solution α-Li_2_SO_4_(ss) (cubic structure). For the composition Li_2_SO_4_/Na_2_SO_4_ 50/50 the stoichiometric compound LiNaSO_4_ (at the proper temperature) undergoes a phase transition from the low-temperature phase (β-phase, trigonal structure) to the high-temperature phase (α-phase, cubic structure).

In Table 1, the theoretical results for the enthalpy of reaction/transition, volumetric energy density, density, and heat capacity (at the transition temperature) obtained from the theoretical study are reported.

From the theoretical results, the composition corresponding to the stoichiometric compound LiNaSO_4_ (50/50) should be the most energetic one compared to the eutectoid composition (79/21), where, a mixture of Li_2_SO_4_ and LiNaSO_4_ is expected at room temperature.

### 3.2. Experimental Results and Discussion

**Figure 2:**
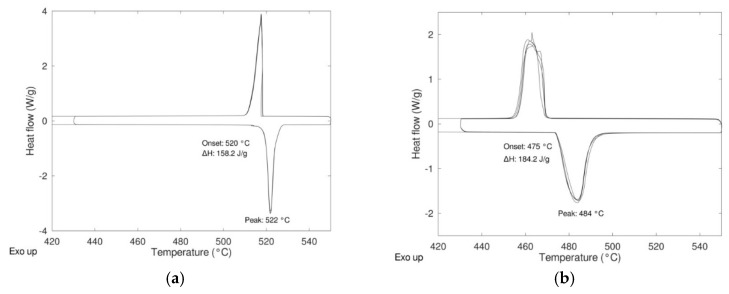
Differential scanning calorimetry **(**DSC) results of the samples (**a**) Li_2_SO_4_/Na_2_SO_4_ 50/50 and (**b**) Li_2_SO_4_/Na_2_SO_4_ 79/21.

In Figure 2 the DSC results relevant to the two compositions “as received”, i.e., not subjected to any preliminary ball milling treatment (only intermixing for 15 min) are reported. Before to record the DSC signals, all the samples were submitted to a preliminary treatment in the oven (inside the hermetically closed DSC aluminum holders) in order to detect any unwanted side reaction (corrosion) with the holder. All the measurements were carried out with a heating rate of 5 K/min performing three heating/cooling cycles between 430 and 550 °C under controlled atmosphere.

For both compositions, the presence of only one peak during heating and cooling suggests that the materials were prepared properly with the correct stoichiometric ratio, especially in the case of the composition 79/21 where some differences were detected between the experimental values reported in the literature. For both compositions, the energy relevant to the transition is quite high, 158 J/g and 184 J/g for 50/50 and 79/21 compositions, respectively, but lower than the theoretical ones reported in Table 1. In order to determine if the differences between the experimental values and the theoretical ones were due to a not complete reactivity in the solid state, the synthesis of the pure material with different microstructures was carried out with the aim to increase the reactivity in the solid state (see Section 2.2).

The powdered materials obtained after ball milling treatment (for 2, 4, and 8 h + mixing for 15 min) were analyzed by X-ray diffraction analysis in order to study their microstructure and if any reactivity in the solid state was promoted by the mechanochemical treatment, keeping in mind that the goal of the ball milling treatment was only to produce very fine powder (decreasing of the crystallite sizes/increasing the specific surface area) and to guarantee a high dispersion level of the reactants (high intermixing degree) to maximize the reactivity during the subsequent heating treatment. The same analysis was carried out for the two compositions investigated (50/50, 79/21) obtaining similar results.

#### 3.2.1. The Li_2_SO_4_/Na_2_SO_4_ 50/50 System

**Figure 3:**
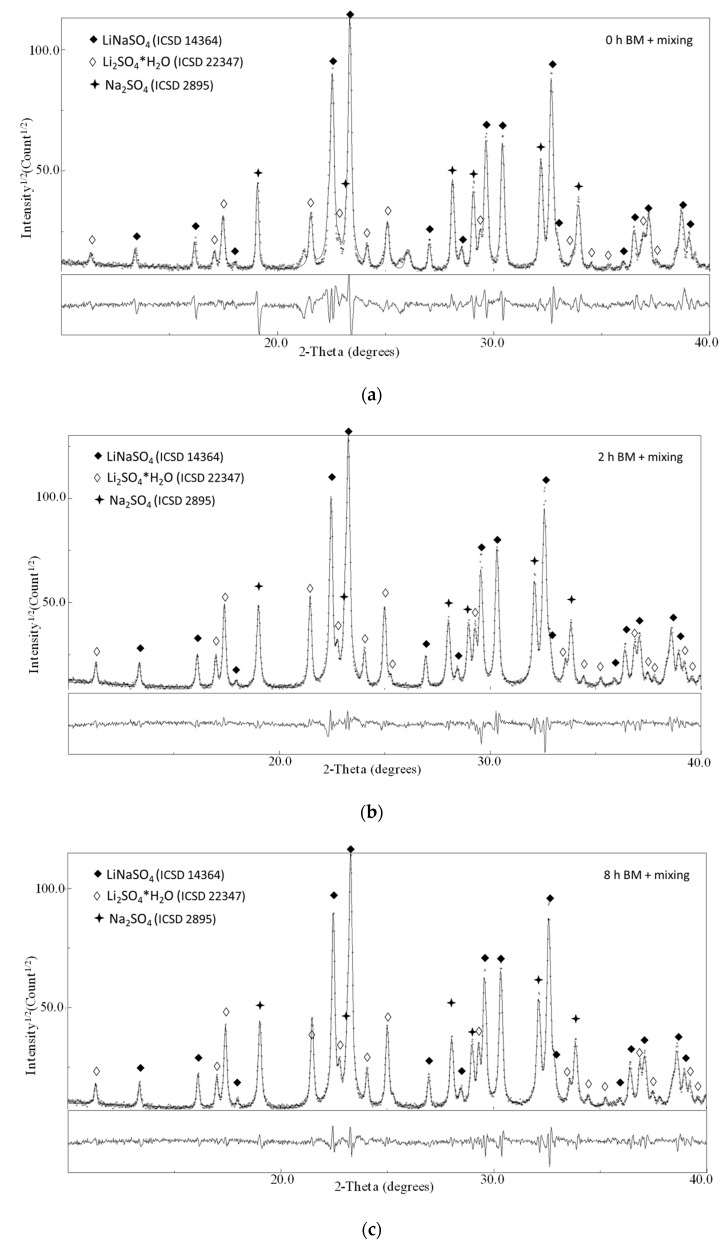
X-ray diffraction patterns of the sample Li_2_SO_4_/Na_2_SO_4_ 50/50 after (**a**) 0 h BM + 15 mixing, (**b**) 2 h BM + 15 mixing and (**c**) 8 h BM + 15 mixing.

In Figure 3, the XRD patterns for the composition 50/50 as a function of the milling time (the pure materials milled 0, 2, and 8 h) are shown.

The ball-milling treatment activates the reaction in the solid state even if, for the intermixing of the pure compounds (milled 0, 2, 4, and 8 h), mild milling conditions were applied. This is confirmed by the partial conversion of the reactant in the stoichiometric phase LiNaSO_4_ (room temperature β-phase ICSD 14364) after 15 min of milling. Moreover, for all the samples, the phases Na_2_SO_4_ (ICSD 2895) and hydrated Li_2_SO_4_∙H_2_O (ICSD 22347) are also detected (reflections corresponding to pure Li_2_SO_4_ (ICSD 2512) can be observed only in the case of the as-received sample). The hydration of Li_2_SO_4_ happens during the XRD exposure due to the long measurement time necessary to obtain high-quality patterns for the refinement procedure. The presence of the hydrated phase affects neither the quantitative analysis to determine the content of LiNaSO_4_ nor the reactivity upon heating.

The activation of the reaction during low energy mechanical mixing was not expected. It is well known that mechanical milling is a powerful technique for material destabilization, increasing the number of defects (grain boundaries, dislocation, etc.) and helping to overcome the energetic barrier activating the transformation in the solid state [32]. The Rietveld refinement analysis of the diffraction profiles of the samples 0, 2, 4 (not shown here), and 8 h BM allowed to determine the structural parameters of all the phases detected after milling (cell parameters, crystallite sizes) and to perform the quantitative analysis (a part in the case of the 0 h samples where the goodness of the fit is not sufficient to obtain reliable results). The results of the fitting procedure together with the corresponding error are reported in Table 2. In the table, the cell parameters (a and b), the crystallite size and the volume fraction relevant to the LiNaSO_4_ phase are reported.

**Figure 4:**
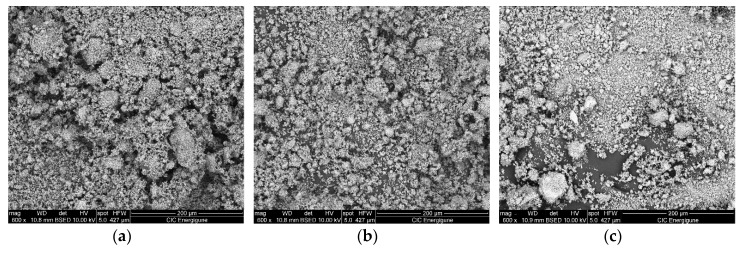
SEM pictures of Li_2_SO_4_/Na_2_SO_4_ 50/50 after (**a**) 0 h BM + 15 min. mixing, (**b**) 2 h BM + 15 min. mixing, (**c**) 8 h BM + 15 min. mixing.

The structural parameters relevant to the LiNaSO_4_ are similar for all the samples studied even if a slight decrease of the cell parameters (a and b) can be observed when going from the sample 0 h BM to the sample 8 h BM. For all the samples, the results show that a considerable amount (between 50 and 60 vol.%) of LiNaSO_4_ is formed for very short milling time in mild conditions. Moreover, the results clearly show that the reaction during milling (Li_2_SO_4_ + Na_2_SO_4_ → LiNaSO_4_) is independent (or very slightly influenced) by the preliminary treatment of the pure materials (milled at high energy for 2, 4, and 8 h). This is probably due to the high reactivity of the systems that need only small energy (in this case, mechanical energy and the thermal contribution due to the local increase of the temperature at each impact) to overcome the energetic barrier of the reaction/transformation. Moreover, the preliminary mechanical treatment of the pure material does not induce major transformation in the microstructure as shown by the SEM pictures shown in Figure 4 where the 50/50 composition is reported as a function of the milling time. This is probably due to the absence of plastic deformation of the materials resulting from the mechanical properties of the salts.

The EDX analysis, performed on all the samples studied, confirms the absence of contamination coming from the milling media (Fe or Ni) that could influence the reactivity upon milling. These results were expected due to the controlled experimental conditions applied during milling in order to prevent the possible contamination coming from the milling media (see Experimental Section).

**Figure 5:**
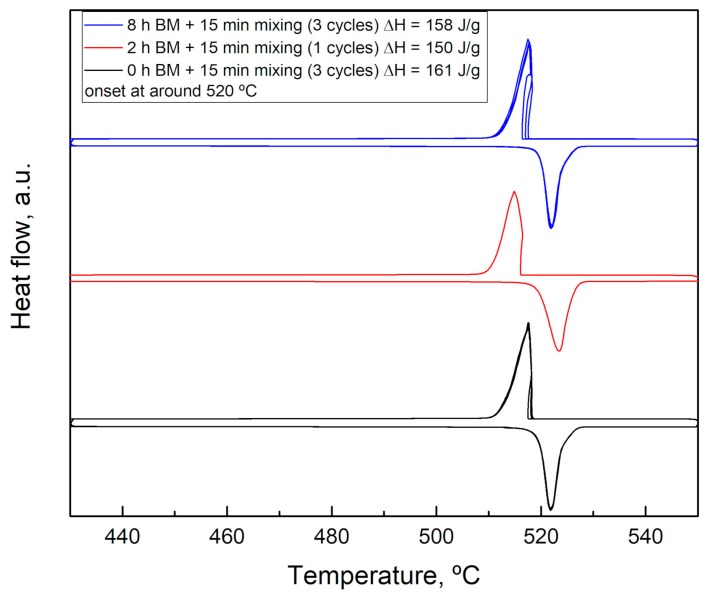
DSC results of the samples Li_2_SO_4_/Na_2_SO_4_ 50/50 milled 0, 2, and 8 h.

The similar nature of the three samples is confirmed by the behavior upon heating. In Figure 5 the DSC signal of the samples milled 0, 2, and 8 h are reported.

The DSC results show that there are no differences in the reactivity of the three samples studied a part a slightly different enthalpy of transformation, probably due to some impurities in the starting materials. This result is very interesting because it confirms the great reactivity of the system, even avoiding any preliminary activation.

This aspect is very important when thinking about the application in thermal energy storage because it contributes to maintaining low costs related to the preparation of the material. Moreover, the high energy involved in the transformation (above 150 J/g) is very promising for TES applications at high temperatures. The thermal energy storage performances of these materials will be discussed at the end of this article.

**Figure 6:**
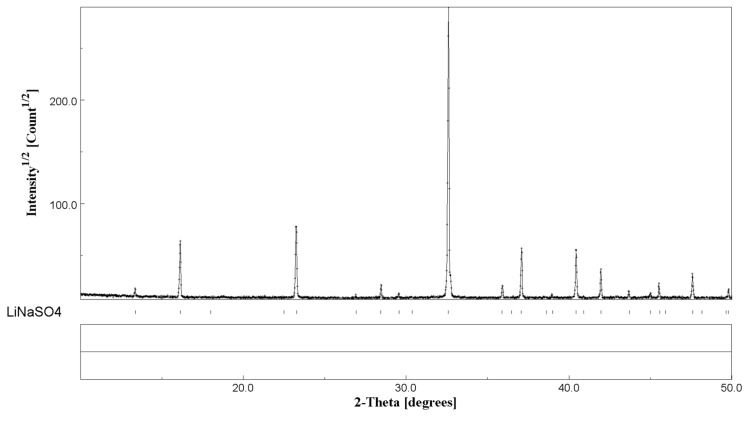
XRD results of Li_2_SO_4_/Na_2_SO_4_ 50/50 after heating treatment.

When comparing the results obtained in this paper with the literature, a discrepancy can be highlighted in the enthalpy of the phase transition. Some authors found higher values, ΔH = 213 J/g [12] and lower or comparable values, ΔH = 130 J/g [33]) and 165.1 J/g) [14]. In this work, the XRD results after the heat treatment (samples 0 h + 15 min. mixing) shows the presence of the pure room temperature β-phase LiNaSO_4_ (see Figure 6). The XRD pattern shows a considerable texture with the absence of some reflections due to the preparation of the sample in the form of a pellet (see Experimental Section). The cell parameters relevant to the LiNaSO_4_ phase are reported in Table 2. The lower values obtained for both parameters a and c indicate a contraction of the crystal structure (lower volume of the elementary unit), probably due to the releasing of the stresses (introduced by the mechanical treatment) during heating.

**Figure 7:**
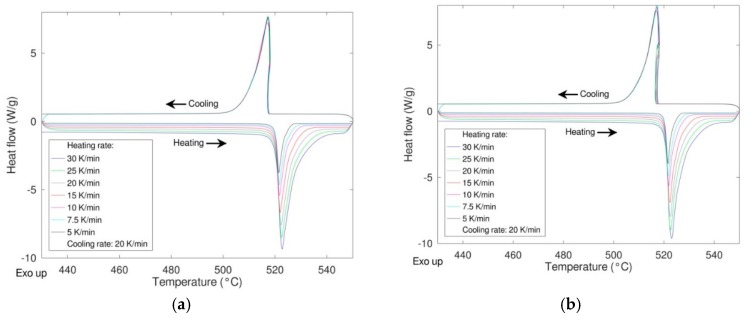
Effect of the heating rate on the phase transition LiNaSO_4_ α ↔ β “as received” (**a**) and milled 8 h (**b**).

In order to study the response of the material under different conditions, the reactivity as a function of the heating rate and the durability when subjected to multiple heating/cooling cycles were studied. In the first case, different heating rates were applied (5, 7.5, 10, 15, 20, 25, 30 K/min) maintaining the cooling rate at 20 K/min. In Figure 6 the DSC results of these experiments are shown for the samples milled 0 and 8 h (see Figure 7).

**Figure 8:**
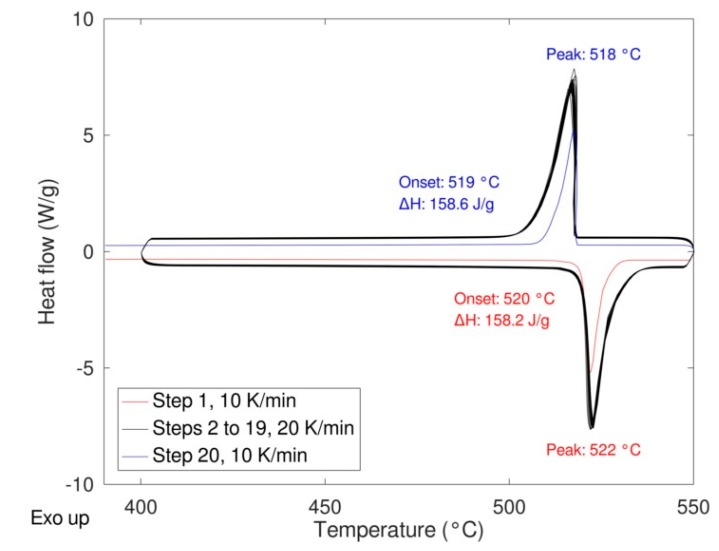
LiNaSO_4_ behavior upon cycling (19 cycles at 20 K/min, 1 cycle at 10 K/min).

As expected, a very similar behavior was obtained for the two samples with no significative differences in the shape of the peaks, thermodynamic values, and transition temperatures (both onset and peak temperature). The same samples were then cycled 20 times at a rate of 20 K/min performing the first and the last cycle at 10 K/min to calculate the enthalpy of the transition at the beginning and at the end of the experiment thus determining the stability upon cycling (Figure 8). In the figure, the enthalpies values relevant to the first and the last cycle are also depicted.

**Figure 9:**
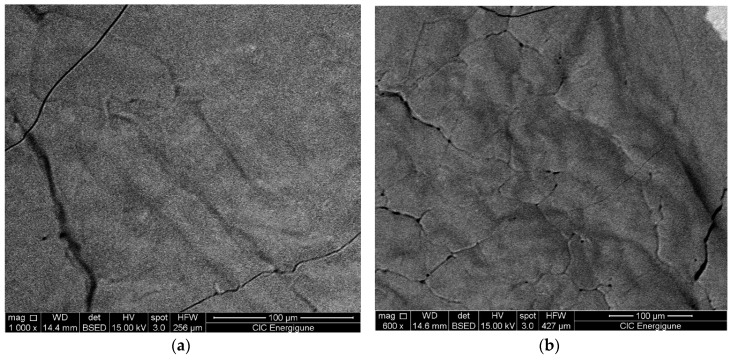
SEM picture of LiNaSO_4_ (**a**) after three cycles in the DSC and (**b**) after twenty cycles in the DSC.

The results are very promising in terms of TES performance. The sample shows high reversibility and fast and good kinetics with a hysteresis below two degrees. Almost no variation of the enthalpy was observed after 20 cycles being the slight difference within the experimental error. The SEM pictures (see Figure 9) after 20 cycles and after three cycles in the DSC show that, after already three cycles, there is the sintering of the material. It is worth to note that the sintering does not affect the reactivity.

The last experiment we want to report in this paper is the behavior of the materials in air. Indeed, the utilization of the materials for TES application in ambient conditions (air contact) is highly desirable. This would allow the use (for example) of air as heat transfer fluid in direct contact with the material with no special care to avoid contamination.

**Figure 10:**
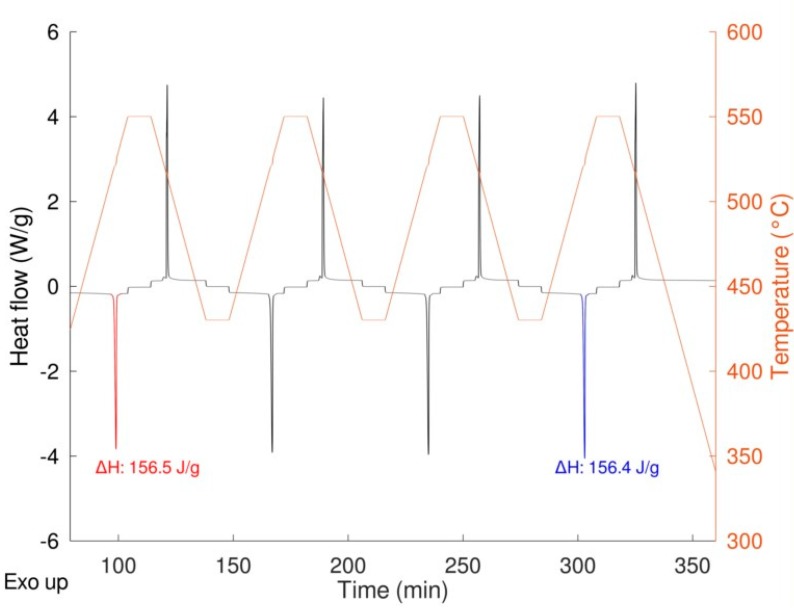
LiNaSO_4_ behavior in air.

The material was prepared in an oven with the pellet placed above an Al_2_O_3_ support (see Section 2.3). The results confirm the possibility to work in air without any degradation of the sample after three cycles (Figure 10). It is worth to note that there is a slight variation from the stoichiometric composition as shown by the presence of an extra small peak in the DSC, well visible in the cooling step. At this moment, aging tests are being carried out in order to determine the performance of the material after a representative number of cycles (at least 100).

#### 3.2.2. The Li_2_SO_4_/Na_2_SO_4_ 79/21 System

**Figure 11:**
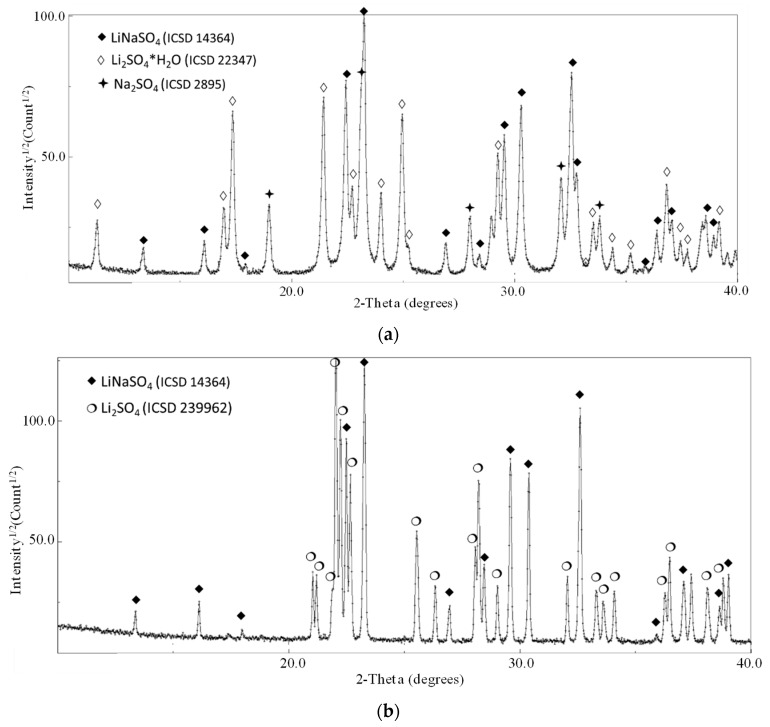
XRD results of Li_2_SO_4_/Na_2_SO_4_ 79/21 (0 h BM + 15 min mixing) (**a**) after milling and (**b**) after heating treatment (in air).

The eutectoid composition 79/21 was studied under similar conditions to those applied to the 50/50 composition. Comparable results were obtained regarding the reactivity after milling and subsequent thermodynamic characterization. As an example, in Figure 11, the XRD results of the sample 0 h + 15 min. of mixing prepared (a) after milling and (b) after heating in the oven under air atmosphere are reported. The milling applied for the preliminary preparation of the material induced the formation of the stoichiometric phase LiNaSO_4_. The refinement results (not shown here) of the sample after milling, confirm the presence of around 60% of this phase together with Na_2_SO_4_, hydrated Li_2_SO_4_∙H_2_O and traces of Li_2_SO_4_. The XRD of the sample after heating treatment in the oven shows, as expected, only the phases LiNaSO_4_ and anhydrous Li_2_SO_4_ (β phase).

**Figure 12:**
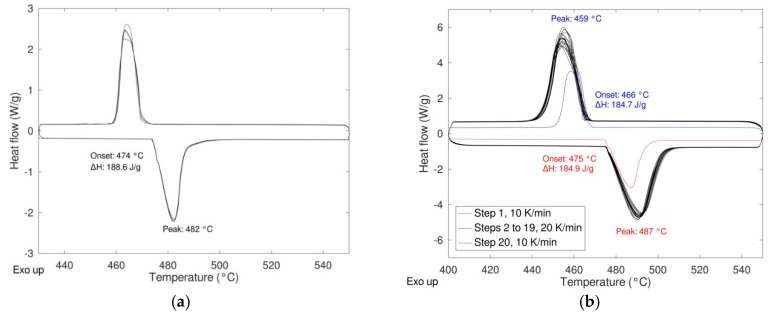
DSC results of Li_2_SO_4_/Na_2_SO_4_ 79/21 (0 h BM + 15 min mixing) (**a**) after milling and (**b**) upon cycling (20 cycles).

The energy related to the eutectoid reaction of the sample prepared in air is reported in Figure 12 where the DSC after preparation and the cycling behavior (20 cycles) are shown. The DSC results show the high reproducibility of the reaction. The enthalpy after 20 cycles is 185 J/g.

As expected from the theoretical results (Table 1) the onset of the reaction is at around 470 °C. It is noteworthy that the enthalpy associated with the reaction is higher when compared to the composition 50/50 (184 J/g against 160 J/g). As already pointed out, this is in contrast with the theoretical prediction where the formation of the stoichiometric phase should be characterized by a higher enthalpy than in the eutectic composition (318 J/g against 270 J/g). For the moment we do not have an exhaustive explanation for this behaviour, as it is not clear why lower values of enthalpies are obtained. Indeed, analysing the material after heating treatment (both compositions) it is undoubtable that, for example, in the case of the 50/50 composition there is the quantitative formation of LiNaSO_4_. These results were reproduced many times arriving always at the same conclusion. For the eutectic composition, similar reasoning can be made observing, after heating treatment, the expected phases in the predicted composition.

### 3.3. Thermal Energy Storage Performance

In Table 3, the TES performance of the two compositions proposed in this study is compared with the reference’s materials in both latent heat storage (NaNO_3_) and sensible storage.

The specific heat reported in the table (both compositions) was measured in this study by DSC analysis. When considering the latent heat, similar TES performances to NaNO_3_ can be achieved, however, we should keep in mind all the additional advantages due to the solid nature of the system. When the sensible storage performance is taken into account it can highlight the considerably higher volumetric energy density in the case of the materials proposed in this paper, calculated using as ΔH the sum of the enthalpy of transition/reaction and the specific heat contribution for a ΔT of 100 °C.

The work is now in progress in order to clarify the discrepancies obtained between the theoretical prediction and the experimental results. Moreover, experiments using a representative amount of materials (grams) are ongoing together with the study of the mechanical properties in view of, for example, a packed bed TES application.

## 4. Conclusions

The Li_2_SO_4_–Na_2_SO_4_ system is very promising for thermal energy storage applications at high temperatures. The high enthalpy of reaction/transformation, the good reversible behaviour, the fast kinetics, the negligible hysteresis, the possibility to work in air and the solid nature make it a very promising material for the application addressed. Because of the nature of this system (phase transition and solid-state nature) it could be used as latent heat storage materials in applications that require constant temperature and as sensible heat storage material in a range of temperature.

## Figures and Tables

**Table 1:** Theoretical results for the compositions Li_2_SO_4_/Na_2_SO_4_ 50/50 and 79/21.

Li_2_SO_4_/Na_2_SO_4_	Temperature °C	ΔH kJ/mol	Volumetric Energy Density (kWh/ m^3^)	Density kg/m^3^	Cp kJ/kg K
50/50	515	40.080 (318 J/g)	217	2458	1.72
79/21	470	31.530 (270 J/g)	173	2309	1.58

**Table 2:** Results of the fitting procedure for the samples Li_2_SO_4_/Na_2_SO_4_ 50/50 (the pure materials milled 0, 2, and 8 h and then mixed for 15 min in the right composition).

Li_2_SO_4_/Na_2_SO_4_ 50/50	LiNaSO_4_ a (nm)	LiNaSO_4_ c (nm)	LiNaSO_4_ <d> (nm)	LiNaSO_4_ Volume Fraction
0 h BM + 15 min mixing	0.7640 ± 1.14 × 10^−5^	0.9874 ± 2.51 × 10^−5^	169.8 ± 2.46	–
2 h BM + 15 min mixing	0.7640 ± 1.14 × 10^−5^	0.9873 ± 2.51 × 10^−5^	189.2 ± 2.92	0.5982 ± 0.0117
8 h BM + 15 min mixing	0.7636 ± 1.32 × 10^−5^	0.9868 ± 2.76 × 10^−5^	176.7 ± 3.30	0.6134 ± 0.0208
0 h + mix after heating	0.7634 ± 7.58 × 10^−4^	0.9851 ± 1.16 × 10^−3^	–	–

**Table 3:** Li_2_SO_4_/Na_2_SO_4_ TES performance. Comparison of sensible and latent heat storage.

**Latent**	**Li_2_SO_4_/Na_2_SO_4_**	**ΔH** **Reaction/Transition** **J/g**	**Cp** **J/g.K**	**Density** **(Theoretical)** **kg/m^3^**	**Volumetric Energy** **Density** **kWh/m^3^**
	50/50	160	2.19	2458	109
	79/21	185	1.6	2309	118
	NaNO_3_[34]	177	1.7	2.26	111
**Sensible**	**Li_2_SO_4_/Na_2_SO_4_**	**ΔH** **Reaction/Transition** **J/g**	**Cp** **J/g.K**	**Cp**Δ**T** **(ΔT = 100 K)** **J/g**	**Total Volumetric Energy** **Density** **kWh/m^3^**
	50/50	160	2.19	219	258
	79/21	185	1.6	160	221
	Sand Rock [34]		1.3	130	60
	Reinforced Concrete [34]		0.85	58	50
	Magnetite [35]		0.9	100	120
